# Potential Effect of CD271 on Human Mesenchymal Stromal Cell Proliferation and Differentiation

**DOI:** 10.3390/ijms160715609

**Published:** 2015-07-09

**Authors:** Giovanna Calabrese, Raffaella Giuffrida, Debora Lo Furno, Nunziatina Laura Parrinello, Stefano Forte, Rosario Gulino, Cristina Colarossi, Luciana Rita Schinocca, Rosario Giuffrida, Venera Cardile, Lorenzo Memeo

**Affiliations:** 1 IOM Ricerca, 95029 Viagrande, Italy; E-Mails: giovanna.calabrese@grupposamed.com (G.C.); raffaella.giuffrida@grupposamed.com (Ra.G.); rosario.gulino@grupposamed.com or rogulino@unict.it (Ro.G.); 2 Department of Biomedical and Biotechnological Sciences, Physiology Section, University of Catania, 95125 Catania, Italy; E-Mails: lofurno@unict.it (D.L.F.); giuffros@unict.it (R.G.); cardile@unict.it (V.C.); 3 Department of Experimental Oncology, Mediterranean Institute of Oncology, 95029 Viagrande, Italy; E-Mails: lauraparrinello@tiscali.it (N.L.P.); cristina.colarossi@grupposamed.com (C.C.); luciana.schinocca@grupposamed.com (L.R.S.); 4 Department of Biomedical Sciences, Hematology Section, University of Catania, 95124 Catania, Italy

**Keywords:** mesenchymal stromal cells, bone marrow, adipose tissue, adipogenic differentiation, chondrogenic differentiation, osteogenic differentiation, CD271

## Abstract

The Low-Affinity Nerve Growth Factor Receptor (LNGFR), also known as CD271, is a member of the tumor necrosis factor receptor superfamily. The CD271 cell surface marker defines a subset of multipotential mesenchymal stromal cells and may be used to isolate and enrich cells derived from bone marrow aspirate. In this study, we compare the proliferative and differentiation potentials of CD271^+^ and CD271^−^ mesenchymal stromal cells. Mesenchymal stromal cells were isolated from bone marrow aspirate and adipose tissue by plastic adherence and positive selection. The proliferation and differentiation potentials of CD271^+^ and CD271^−^ mesenchymal stromal cells were assessed by inducing osteogenic, adipogenic and chondrogenic *in vitro* differentiation. Compared to CD271^+^, CD271^−^ mesenchymal stromal cells showed a lower proliferation rate and a decreased ability to give rise to osteocytes, adipocytes and chondrocytes. Furthermore, we observed that CD271^+^ mesenchymal stromal cells isolated from adipose tissue displayed a higher efficiency of proliferation and trilineage differentiation compared to CD271^+^ mesenchymal stromal cells isolated from bone marrow samples, although the CD271 expression levels were comparable. In conclusion, these data show that both the presence of CD271 antigen and the source of mesenchymal stromal cells represent important factors in determining the ability of the cells to proliferate and differentiate.

## 1. Introduction

Mesenchymal stem cells (MSCs), also known as multipotent stromal cells or mesenchymal stromal cells, are non-hematopoietic progenitor cells that have been originally identified in the bone marrow [1]. Subsequently, cells with MSC-like characteristic have been identified from a variety of tissues including adipose tissue [2,3,4], fetal liver, lung, spleen [5,6], amniotic fluid [7], cord blood [8], umbilical cord [9,10,11,12], placenta [13,14,15], human endometrium [16,17], and dental pulp [18,19]. MSCs can be isolated either by plastic adherence or by immunomagnetic positive selection. These cells, under appropriate conditions, can give rise to several cell types, including bone, cartilage and fat precursors [20].

CD271 is a neurotrophin receptor and is a member of the tumor necrosis factor receptor superfamily. It is also known as the low-affinity nerve growth factor receptor (NGFR) or p75NTR. CD271 is an ~75 kDa type 1 transmembrane glycophosphoprotein that interacts with neurotrophins such as NGF, brain-derived neurotrophic factor (BDNF), neurotrophin-3 (NT3) and neurotrophin-4 (NT4), which represent a family of growth factors that stimulate neuronal cells to survive and differentiate [21]. CD271 has been found to be expressed in several cell types including neurons, Schwann cells, mesenchymal stem and stromal cells, follicular dendritic cells, melanocytes and numerous cell lines. In the nervous system, it has critical functions in survival [22], differentiation [23] and migration [24] of neuronal cells. Recently, this antigen has been identified as a marker of tumor initiating cells in human melanoma [25,26], esophageal carcinoma [27,28], hypopharyngeal carcinoma [29] and head and neck squamous cell carcinoma [30] where it can modulate different functions such as cell survival and proliferation.

In the present work, we hypothesized that CD271 surface antigen might be able to promote cell proliferation and differentiation along three mesenchymal lineages (adipogenic, osteogenic and chondrogenic)*.* Since, it is already known in the literature that the proliferation and differentiation potential of CD271^+^ bone marrow-derived MSCs is higher than that of corresponding unselected cell lines [31], in this work we decided to compare bone marrow and adipose-derived CD271 positive MSCs both differently selected by adherence and immunomagnetic selection*.* For this purpose we have analysed twelve MSC lines derived from two different sources, bone marrow (bm) and adipose tissue (ad) expressing low and high CD271 levels (respectively indicated as CD271^−^ and CD271^+^), and in particular 3 bmMSC^CD271−^, 3 bmMSC^CD271+^, 3 adMSC^CD271+^ and 3 adMSCsel^CD271+^ cell lines.

In order to test the hypothesis that CD271 may affect the proliferative and differentiation capabilities of MSCs, cells were cultured in specific media in the presence of inducing factors. The proliferative potential was evaluated by 4′,6-diamidino-2-phenylindole (DAPI) staining and trypan blue test, whereas osteogenesis, chondrogenesis and adipogenesis capabilities were evaluated respectively by Alizarin Red S, Alcian Blue and Oil-Red O stainings. Our findings support the hypothesis that cells with high CD271 expression have greater proliferative and differentiation potentials than CD271 negative cells. Moreover, we found that the source of MSCs represent a further crucial factor for the selection of cells with high proliferative and differentiation potentials. These results could be relevant for the selection of MSCs to be used in the field of regenerative medicine.

## 2. Results and Discussion

### 2.1. Phenotypic Characterization of Mesenchymal Stem Cells (MSCs)

MSCs were isolated either from bone marrow and adipose tissue samples by adhesion to plastic support and CD271 immunopositive selection.

Phenotypical characterization of MSCs was carried out by flow cytometry and immunocytochemistry analysis. Twelve different MSC lines were used to study the expression of typically positive (CD105, CD90, CD73, CD271) and negative (CD45, CD31, CD34) surface markers of stemness.

Flow cytometry results are shown in Table 1. It is worth noting that the expression of negative markers (CD45, CD31, CD34) was not detectable in any cell line, whereas all of them were positive for stem cell markers, such as CD73, CD90 and CD105. Notably, CD271 was the only marker showing statistically significant differences in its expression among the different MSCs types. Pairwise comparison of CD271 expression levels indicates statistically significant differences between bmMSC^CD271−^ and bmMSC^CD271+^, between bmMSC^CD271−^ and adMSC^CD271+^, as well as between bmMSC^CD271−^ and adMSCsel^CD271+^ cell lines.

**Table 1:** Flow cytometry results showing the percentage of cells expressing typical markers of mesenchymal stem cells.

Marker	bmMSC^CD271−^ (Mean ± s.e.m.)	bmMSC^CD271+^ (Mean ± s.e.m.)	adMSC^CD271+^ (Mean ± s.e.m.)	adMSCsel^CD271+^ (Mean ± s.e.m.)	One-Way ANOVA	
					*p*	*p*-Corrected
**CD31**	2.45 ± 0.55	7.08 ± 3.74	2.23 ± 0.92	5.13 ± 2.09	0.403	1.000
**CD45**	2.30 ± 0.60	4.21 ± 1.67	2.93 ± 2.25	2.4 ± 1.6	0.824	1.000
**CD34**	3.44 ± 2.10	5.17 ± 2.28	3.33 ± 1.94	3.83 ± 2.63	0.933	1.000
**CD90**	88.13 ± 0.79	96.10 ± 2.41	87.00 ± 6.21	92.3 ± 6.17	0.513	1.000
**CD73**	89.30 ± 2.72	82.33 ± 6.08	95.10 ± 1.75	94.13 ± 0.37	0.104	1.000
**CD105**	88.10 ± 2.72	85.85 ± 6.08	88.43 ± 1.75	91.93 ± 1.68	0.77	1.000
**CD271**	29.13 ± 8.18	78.83 ± 10.89	89.20 ± 5.66	90.57 ± 4.16	0.001 **	0.01 **
Tukey’s *post-hoc*						
**Sample Types**			**diff**	**ler**	**upr**	***p***
adMSCsel^CD271+^ *vs.* adMSC^CD271+^			1.366667	−33.32805	36.06138	0.999
bmMSC^CD271−^ *vs.* adMSC^CD271+^			−60.066667	−94.76138	−25.37195	0.002 **
bmMSC^CD271+^ *vs.* adMSC^CD271+^			−10.373333	−45.06805	24.32138	0.776
bmMSC^CD271−^ *vs.* adMSCsel^CD271+^			−61.433333	−96.12805	−26.73862	0.002 **
bmMSC^CD271+^ *vs.* adMSCsel^CD271+^			−11.740000	−46.43471	22.95471	0.708
bmMSC^CD271+^ *vs.* bmMSC^CD271−^			49.693333	14.99862	84.38805	0.007 **

Immunopositive cellular fractions for several surface marker assessed by flow cytometry are shown for each cell type. In the top table the statistical significance of percentages differences among the three groups, assessed by one-way ANOVA, is reported in the last two columns (raw and Bonferroni corrected *p* values respectively). CD271 is the only surface marker that shows statistically significant differences. *Post-hoc* pairwise comparisons of CD271 percentages, performed using Tukey’s method, is summarized in the bottom table. Significant differences are observed only between bmMSC^CD271−^ and bmMSC^CD271+^, between bmMSC^CD271−^ and adMSC^CD271+^, as well as between bmMSC^CD271−^ and adMSCsel^CD271+^. ** *p* < 0.01.

**Figure 1:**
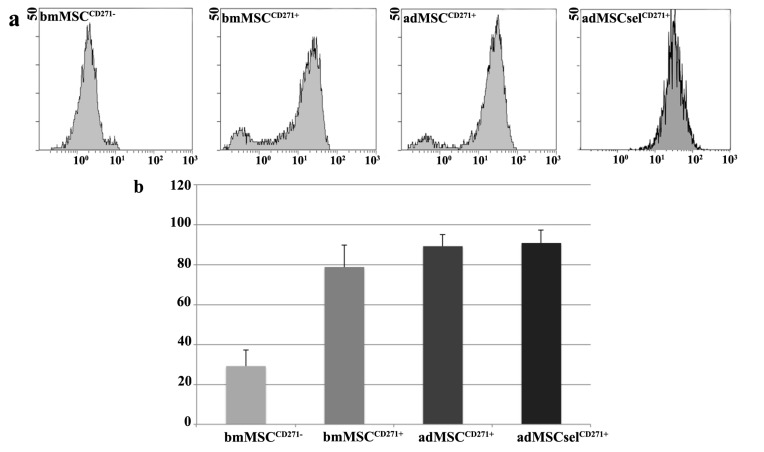
Flow cytometry expression of CD271 in bmMSC^CD271−^, bmMSC^CD271+^, adMSC^CD271+^ and adMSCsel^CD271+^ lines; (**a**) Representative histograms of only one for each type of the four different cell lines; (**b**) Average Percentage of CD271^+^ cells in the four different cell types.

MSCs isolated from bone marrow by adhesion to plastic were weakly positive for CD271 (29%), whereas the percentage of positive cells increased after enrichment by immunomagnetic positive selection (78%) (Figure 1a,b). On the contrary, MSCs derived from adipose tissue already showed higher expression for the same antigen, both by adhesion to plastic support (90%) and immunomagnetic positive selection (91%). Since in the latter case the two cell lines presented comparable CD271 levels, for convenience, all further experiments were performed only on adMSCs isolated by plastic adhesion.

Phenotypical characterization by immunohistochemistry performed in the same cell lines showed results consistent with those obtained by flow cytometry. All cell lines were strongly positive for CD73, CD90 and CD105, whereas the expression of CD45, CD31 and CD34 was not detectable. In addition, bmMSC^CD271−^ was weakly positive for CD271 whereas its expression increased after enrichment by immunomagnetic positive selection. On the other hand, adMSC isolated either by adherence and immunomagnetic selection showed similar high expression of CD271 marker.

Several researchers have reported that CD271 antigen defines a subset of mesenchymal stromal cells and can be used to identify MSCs from different sources [32,33]. Our *in vitro* studies have shown that MSCs, isolated from adipose tissue and bone marrow aspirate by plastic adherence and immunopositive selection, exhibited the same phenotype when characterised by flow cytometry and immunocytochemistry analysis according to published data [34,35]. The only phenotypical difference we found between the differently derived MSCs studied in this work, was represented by the expression of the surface antigen CD271.

### 2.2. The Expression of CD271 by MSCs Could Increase Proliferative and Trilineage Differentiation Potential

In order to assess the proliferative capacity of MSCs, cells were plated at the same density (3.1 × 10^3^ cells/cm^2^) in parallel cultures using the culture conditions described in the experimental section. The proliferative ability of MSCs was assessed both during expansion and differentiation (by trypan blue test and DAPI staining, respectively).

**Figure 2:**
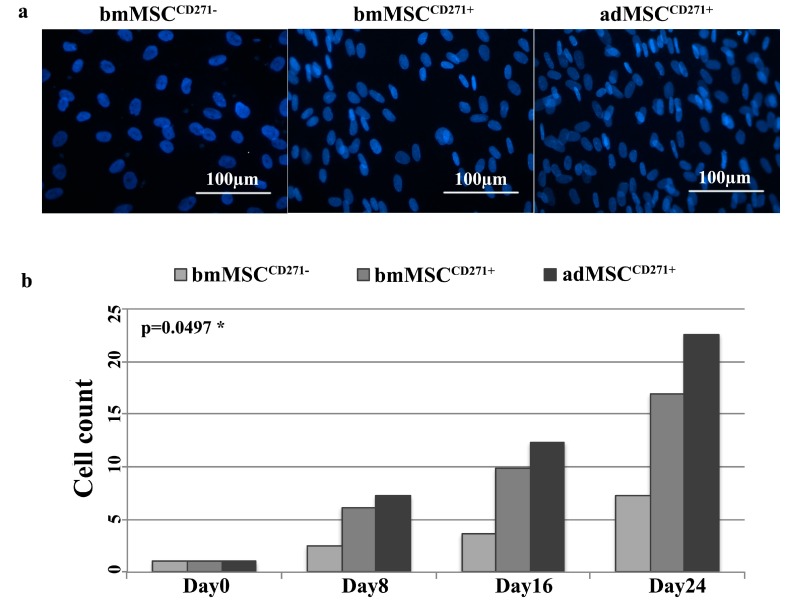
DAPI staining showing the cell density at different time points after osteogenic differentiation. (**a**) Representative DAPI staining of bmMSC^CD271−^, bmMSC^CD271+^ and adMSC^CD271^^+^ at the 24th day of differentiation; (**b**) Similarly, cell count shows statistically significant differences among cell lines at day 8, 16 and 24 (*p* = 0.0497). In particular, data clearly show that CD271^+^ MSCs have a higher proliferation rate as compared to CD271^−^ MSCs, with statistical significance maintained in adMSC^CD271^^+^ *vs.* bmMSC^CD271−^ pairwise comparison (*p* = 0.0381). * *p* < 0.05.

Our results show that over a period of 24 days of osteogenic induction, the proliferation rate of bmMSC^CD271+^ and adMSC^CD271^^+^ was considerably higher than that of bmMSC^CD271−^ (Figure 2a,b).

Trypan blue test indicated that, from 0 to 72 h after seeding, the proliferation rate of bmMSC^CD271+^ and adMSC^CD271+^ was considerably greater than that observed in bmMSC^CD271−^, in particular we found that the proliferation rate of bmMSC^CD271+^ was 2.5-fold higher than bmMSC^CD271−^, while adMSC^CD271+^ was 3-fold higher than bmMSC^CD271−^, and finally, adMSC^CD271+^ presented a 1.2-fold higher proliferation rate compared to bmMSC^CD271+^.

To determine the differentiation capacity towards osteogenic, chondrogenic and adipogenic lineage, cells at passage 2–3 were cultured for 24–28 days in tissue-specific media. Our data show that both CD271^+^ and CD271^−^-MSCs were able to differentiate into these three lineages as revealed by specific staining. The cells that underwent differentiation showed morphological changes under the influence of specific inducing factors. This was revealed by accumulation of neutral lipid vacuoles during adipogenesis, whereas the occurrence of calcium stores in extracellular matrix was found during osteogenic differentiation. On the other hand, the formation of glycosaminoglycans (GAGs) in the matrix has been found during chondrogenesis.

**Figure 3:**
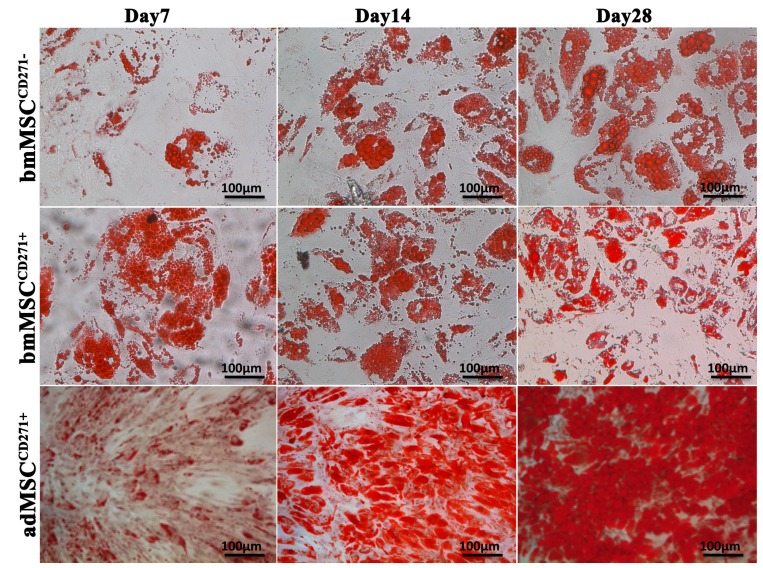
Oil-Red O staining of bmMSC^CD271−^, bmMSC^CD271+^ and adMSC^CD271+^ after 7, 14 and 28 days of adipogenic differentiation.

In particular, after seven days of culture with adipogenic medium, all cell lines showed a good level of proliferation, though CD271^+^-MSCs appeared more numerous. In both cases, morphological changes were observed. In fact, a population of larger rounded cells presenting numerous fat vacuoles in the cytoplasm was easily detectable. Their nature as adipose cells was assessed by Oil-Red O staining (Figure 3). By comparing the three cell lineages, it appears that adipose cells coming from adMSC^CD271+^ are more numerous than those derived from bmMSC^CD271+^ or bmMSC^CD271−^. After 14 days, a high rate of proliferation was observed in all control cultures growing in basal medium without inducing factors, but the densest populations have been observed in adMSC^CD271+^ cultures. Moreover, adMSC^CD271+^ grown in conditioned cultures displayed larger and more numerous vacuoles in their cytoplasm.

This trend was confirmed even at later stages of differentiation. In fact, larger cells and bigger vacuoles were observed at 28 days, always more evidently for adMSC^CD271+^ cultures.

**Figure 4:**
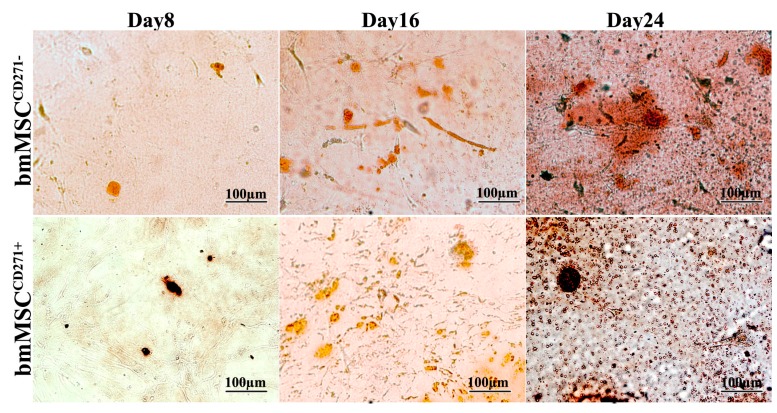

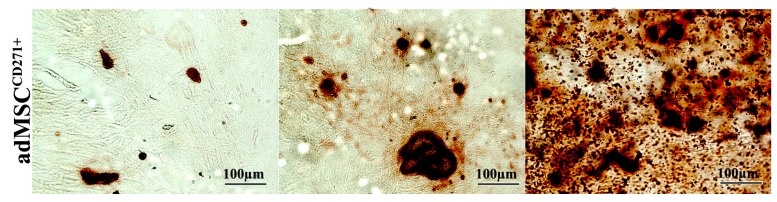
Alizarin Red staining of bmMSC^CD271−^, bmMSC^CD271+^ and adMSC^CD271+^ after 8, 16 and 24 days of osteogenic induction.

Cells growing in osteogenic medium produced after eight days a more mineralized extracellular matrix, resulting in a gradual increase of the calcium stores within the cytoplasm. Alizarin Red staining show that cell calcium content increased with time in all cell lines but more evidently in adMSC^CD271+^, as compared to bmMSC^CD271+^ and bmMSC^CD271−^ (Figure 4). After 16 days of osteogenic induction, adMSC^CD271+^ cultured in conditioned medium displayed a greater number and size of calcium stores in the matrix.

The same behaviour was observed also after 24 days of differentiation. In fact, a greater amount of calcium stores within the cytoplasm was observed, always more markedly in adMSC^CD271+^ cultures.

**Figure 5:**
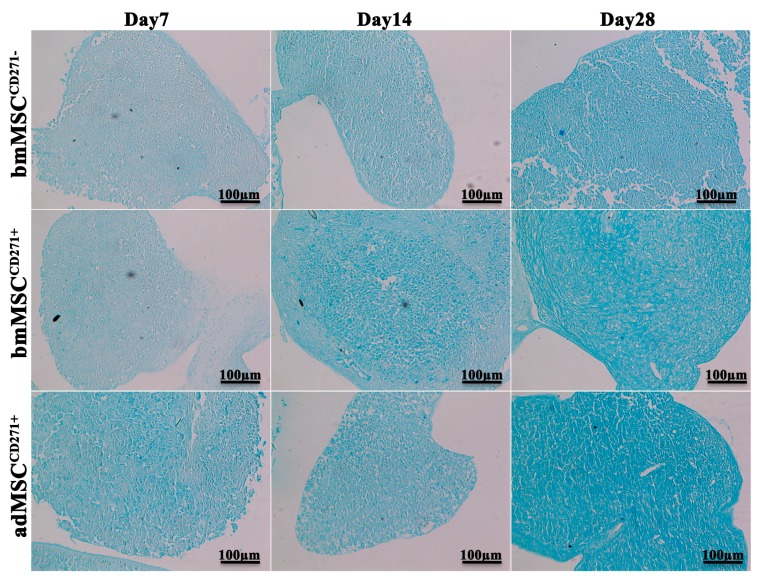
Alcian blue staining of bmMSC^CD271−^, bmMSC^CD271+^ and adMSC^CD271+^ after 7, 14 and 28 days of chondrogenic differentiation.

After seven days of culture in the presence of chondrogenic inducing factors, MSCs began to differentiate as shown by Alcian blue, which stains glycosaminoglycans in cartilages (Figure 5). By comparing the three cell lineages, adMSC^CD271+^ showed a stronger Alcian blue staining compared to bmMSC^CD271+^ and bmMSC^CD271−^, indicating the formation of higher amount of GAGs in extracellular matrix. After 14 and 28 days, all cell cultures displayed a progressive increase of Alcian blue staining, which again appeared stronger in adMSC^CD271+^ cultures compared to bmMSC^CD271+^ and bmMSC^CD271−^.

The present data demonstrate that: (1) MSC^CD271+^ proliferative ability was significantly greater than that observed in MSC^CD271−^; (2) MSC^CD271+^ presented a much stronger adipogenic, osteogenic and chondrogenic potential, when compared to MSC^CD271−^ and (3) both proliferation rate and differentiative ability of adMSC^CD271+^ were more marked than those of bmMSC^CD271+^. These results suggest that: (1) the presence of CD271 could trigger a subset of MSCs to a significant higher proliferation rate and differentiative potentials than the MSCs subset lacking the same antigen; (2) the source of mesenchymal stromal cells could represent an important factor in determining the ability of the cells to proliferate and differentiate. In support of our hypothesis, several recent publications report that MSCs selected for CD271 exhibit a higher proliferative ability compared to CD271 negative cells [36,37]. Furthermore, other studies, conducted in cell systems different from MSCs, have better elucidated the role of CD271 in cell biology. Indeed, it has been reported that CD271 can be present in different cell types and its expression can influence a variety of cell functions such as cell survival, apoptosis, proliferation, migration, differentiation [38,39,40,41,42] and even stem cell tumorigenic potential [43,44]. In addition, many reports in the literature [45,46,47,48] show that adipose tissue represents the best source to isolate MSCs since cells are both readily available in large quantities and exhibit a very high proliferative capability.

## 3. Experimental Section

### 3.1. Isolation, Expansion and Characterization of MSCs Derived from Adipose Tissue/Lipoaspirate and Bone Marrow

Human mesenchymal stem cells were derived from adipose tissue biopsies/lipoaspirates and bone marrow supplied by IOM Spa (Viagrande, Italy) and Cannizzaro Hospital (Catania, Italy), under an approved Institutional Review Board protocol (project ID code: 829_1 of 8 February 2013, IOM Institutional Review Board) and after informed consent. Isolation of MSCs from adipose tissue and bone marrow was performed as previously reported [49,50]. The bone marrow aspirates were diluted with Hank’s Balanced Salt Solution (HBSS), without Ca^2+^ and Mg^2+^ (Invitrogen, part of Thermo Fisher Scientific corporation, Waltham, MA, USA). The resulting solution was gently layered on a previously prepared Ficoll gradient. The buffy coat placed at the HBSS-Ficoll interface was recovered by gentle suction with a pipette Pasteur and diluted with HBSS. After centrifugation, the cell pellet was resuspended in MSC-GM growth medium (MSC-GM BulletKit: MSCBM hMSC basal medium plus MSCGM hMSC SingleQuot Kit; Lonza Group Ltd., Basel, Switzerland). Cells were seeded on culture flask and after 24 h non-adherent cells were removed. Selected MSCs were maintained in culture, medium changed every 3–4 day and cells were expanded until 80%–90% of confluence was reached.

A population enriched in CD271^+^-MSCs was isolated by positive CD271 immunoselection (CD271 MicroBeads Kit-PE human, Miltenyi Biotec Company, Bergisch Gladbach, Germany) according to manufacturer’s instructions.

In order to distinguish MSCs from hematopoietic stem cells, cells at the first passage were characterized by immunocytochemistry and flow cytometry analysis using several cell surface markers.

**Table 2:** Table summarizing the cell origin and the clinical data of donor patients.

Donor	Age	Sex	Sources	Disease	Diagnosis	Follow-up
**BM_1**	66	M	Aspirate	CHL	Post-chemiotherapy	Healthy
**BM_2**	70	M	Aspirate	CML	Post-chemiotherapy	Healthy
**BM_3**	64	F	Aspirate	CML	Onset	Healthy
**AD_1**	62	M	Kidney	–	No chemiotherapy	Healthy
**AD_2**	61	F	Breast	–	No chemiotherapy	Healthy
**AD_3**	60	F	Colon	–	No chemiotherapy	Healthy

CHL, Classical Hodgkin’s Lymphoma; CML, Chronic Myelogenous Leukemia.

Immunocytochemistry was performed on cells seeded in 8-well BD Falcon culture slides at a density of 5000 cells per cm^2^ in MSC-GM (Lonza, Basel, Switzerland). After fixation with 4% Paraformaldehyde (PFA), cells were permeabilized in 0.4% Triton and then blocked by incubation in 5% Donkey serum for 1 h. The primary incubation was performed, overnight at 4 °C, with the following anti-human antibodies: mouse CD105 (1:50, Novus Biologicals, Littleton, CO, USA), mouse CD90 (1:50, Santa Cruz Biotechnology, Dallas, TX, USA), mouse CD73 (1:25, Novus Biologicals), rabbit CD45 (1:100, Epitomics, Burlingame, CA, USA), rabbit CD34 (1:100, Epitomics), mouse CD31 (1:100, Santa Cruz Biotechnology), mouse CD117 (1:100, Abnova, Walnut, CA, USA). After washing, slides were incubated with the appropriate secondary AlexaFluor 568 antibodies (Life Technologies Italia, Monza, Italy) at the dilution of 1:2000 for 2 h at RT. Nuclei were counterstained with DAPI (1:5000) for 5 min. Finally, slides were mounted in fluorescent mounting medium Permafluor (Thermo Scientific, Waltham, MA, USA) and digital images were acquired using a Leica DMI4000B fluorescence microscope (Leica, Wetzlar, Germany).

For flow cytometry analysis, cells were detached with 0.05% trypsin/EDTA and washed in PBS. 1 × 10^4^ cells/tube were stained with the following antibodies: CD45 FITC (Clone J.33), CD34 PE (Clone 581), CD73 PE (Clone 581), CD90 FITC (Clone F15.42.1.5), CD105 PE (Clone 1G2), CD31PE (Clone 1F11), CD271 FITC (Clone ME20.4-1.H4) and corresponding isotypic controls according to manufacturer indications. All antibodies were purchased from Beckman Coulter (Milano, Italy), except CD271 that was provided by Miltenyi Biotec (Bologna, Italy).

All tubes were incubated in the dark for 20 min at room temperature. Cells were then washed with PBS and finally analysed by flow cytometry using an FC-500 five-color flow cytometer (Beckman Coulter, Pasadena, CA, USA). For each tube, 1000 events were acquired. CXP Analysis software (Beckman Coulter©, Inc.) was used for data analysis.

Percentages of CD31, CD45, CD34, CD90, CD73, CD105 and CD271 positive cells where compared in bmMSC^CD271−^, bmMSC^CD271+^, adMSC^CD271+^ and adMSCsel^CD271+^ by analysis of variance [51] using R statistical environment [52]. The resulting *p* values were corrected using Bonferroni correction to minimize false positive arising from multiple testing and epitopes that showed *p* < 0.05 in the inferential test where also evaluated *post-hoc* using Tukey Honest Significant Differences [53].

### 3.2. Proliferative and Trilineage Differentiation Potential of CD271^+^-MSCs and CD271^−^-MSCs. Proliferation Assay Was Carried out by DAPI and Trypan Blue Staining

For DAPI staining, after osteogenic differentiation the cells were fixed in 4% PFA for 15 min and permeabilized in 0.3% Triton X-100 for 5 min. The cells were washed 3 times with PBS and the nuclei counterstained with DAPI (1:5000) in PBS for 5 min. Slides were mounted in fluorescent mounting medium Permafluor (Thermo Scientific) and digital images were acquired using a Leica DMI4000B fluorescence microscope. At least five images from each sample were taken for the count and the experiments were repeated at least four times. The average score obtained from all experiments is reported as total cell number.

Proliferation rates differences were assessed using Friedman rank sum test with Wilcoxon-Nemenyi-McDonald-Thompson test as *post-hoc* for pairwise comparisons.

For trypan blue test, 5000 cell/cm^2^ were plated in growth medium and incubated at 37 °C in a humidified atmosphere containing 5% CO_2_. After 24, 48 and 72 h, the cells were detached, stained with trypan blue and counted under microscope.

To induce adipocyte differentiation, 2.1 × 10^4^ MSCs were cultured in adipogenic medium supplemented with differentiation inducing factors (adipogenic differentiation BulletKit medium, Lonza) for 7, 14, 21 and 28 days. Cell cultures were stopped at the assigned time points, with 10% PFA for 10 min. Then, cells were stained with fresh Oil Red-O solution (Sigma-Aldrich, Saint Louis, MO, USA) according to the manufacturer protocol. The total number of oil red positive adipocytes or adipocyte colonies in each flask was counted.

For the induction of osteogenic differentiation, MSCs were seeded at a density of 3.1 × 10^4^ cells/cm^2^ on collagen I (Serva, Heidelberg, Germany) coated plate in expansion medium at 37 °C, in a humidified atmosphere of 5% CO_2_. After 24 h, the medium was removed and replaced with MSC-GM medium supplemented with osteogenic differentiation promoting factors (hMSC osteogenic differentiation BulletKit, Lonza). The growth medium was completely replaced every 3–4 days with fresh medium. The osteogenic differentiation was observed during the whole period by microscopy and stopped on day 24 after induction. The osteogenic phenotype was confirmed by Alizarin Red S staining (Panreac, Castellar del Valles, Barcellona, Spain).

For the staining, an Alizarin Red S solution was prepared according to the manufacturer protocol. The medium was removed from the culture slides and, after washing, cells were fixed with 4% PFA and incubated with Alizarin Red S solution for 5 min.

To induce chondrogenic differentiation, 2.5 × 10^5^ cells were centrifuged to form a three-dimensional aggregate and resuspended in chondrogenic basal medium, containing chondrogenic inducing factors (hMSC chondrogenic differentiation BulletKit, Lonza) and TGF-β3 (Lonza). Pellets were incubated at 37 °C in a humidified atmosphere of 5% CO_2_. The growth medium was completely replaced every 2–3 days with fresh medium. The chondrogenic differentiation was completed on day 28 after induction. Pellets were fixed in formalin and paraffin embedded for histological processing. Thin sections were stained with Alcian Blue (Panreac).

For the staining, an Alcian Blue solution was prepared according to manufacturer protocol. Slides were deparaffined in xylene, re-hydrated through passages in alcoholic solutions and then stained in Alcian Blue solution for 30 min. Slides were finally mounted and examined under light microscope.

## 4. Conclusions

This work was designed to make an *in vitro* comparison of proliferative rate and differentiation potential among three lineages (adipogenic, osteogenic and chondrogenic) of different MSC subtypes. Our results have shown that both the proliferative ability and differential potential of CD271^+^-MSCs was greater than that of CD271^−^-MSCs. Furthermore, MSCs derived from adipose tissue displayed a more evident ability to proliferate and differentiate compared to those derived from bone marrow.

In conclusion, we can suggest that both the presence of CD271 surface antigen and the MSC isolation source might strongly influence the proliferative and differentiation capability of this cell subset. Such evidence would suggest the choice of adMSC^CD271+^ as the most promising cell model for regenerative medicine applications. Further studies are necessary to better understand the cellular mechanisms underlying the functions of CD271 in adipose-derived MSCs.
